# Immaturity-Dependent Hippocampal Neurogenic Promotion and Fate Shift by Low-Dose Propofol in Neonatal Mice Revealed Through Single-Nuclei RNA-Sequencing

**DOI:** 10.3390/biomedicines13112806

**Published:** 2025-11-18

**Authors:** Wen Zhang, Liangtian Lan, Xuanxian Xu, Keyu Chen, Xiaoyu Yang, Xia Feng, Dihan Lu

**Affiliations:** Department of Anesthesiology, The First Affiliated Hospital of Sun Yat-Sen University, No. 2nd Zhongshan Road, Guangzhou 510080, China

**Keywords:** propofol, hippocampal neurogenesis, single-nucleus RNA-seq, neural intermediate progenitor cells, immature pyramidal neurons, granule maturation, neurexin

## Abstract

**Background:** Hippocampal neurogenesis in the dentate gyrus persists into adulthood and plays a crucial role in learning and memory. Early-life exposure to low-dose propofol has been reported to enhance neural development in rodent models, but detailed mechanisms remain unclear. To address this gap, we aimed to investigate how low-dose propofol alters neurogenic lineage differentiation, transcriptional programs, and underlying molecular mechanisms within the early postnatal hippocampal neurogenic niche. **Results**: We conducted an in-depth re-analysis of a published single-nucleus RNA-sequencing (snRNA-seq) dataset from hippocampal tissue of postnatal day 10 (PND10) mice, collected 3 days after low-dose propofol treatment. Uniform Manifold Approximation and Projection (UMAP)-based clustering revealed twelve major cell types, including a population of *Ntng1^+^Fxyd7*^+^*Pcp1*^+^ immature pyramidal neurons (imPYR), lacking the mature markers *Meis2* and *Spock1*. Trajectory analysis revealed two neurogenic lineages (granule and pyramidal) and indicated that propofol biases progenitor fate commitment towards the granule lineage. CellChat analysis demonstrated that propofol enhances Neurexin (Nrxn) signaling to neural progenitor cells, suggesting increased synaptic adhesion and maturation. Differential expression analysis (|log_2_FC| ≥ 0.26, adjusted *p* < 0.01) followed by pathway enrichment revealed that propofol upregulates neurogenic maturation pathways—including synaptogenesis, synaptic transmission, dendritic morphogenesis, and memory-related processes—specifically within neural intermediate progenitor cells (nIPC). **Conclusions:** Together, these findings delineate a coordinated transcriptional and intercellular mechanism by which low-dose propofol reprograms hippocampal neurogenesis during early postnatal development, highlighting progenitor-specific and synapse-oriented processes that may underlie its cognitive-enhancing effects.

## 1. Introduction

Studies have shown that most mammals retain the capacity for neurogenesis in the hippocampus into adulthood [1,2]. This process plays a crucial role in hippocampal neural development and centers on the functionality of neural stem cells (NSCs) that are persistently located in the subgranular zone of the dentate gyrus (DG) [3]. The regulatory mechanisms of postnatal hippocampal neurogenesis are diverse, including intrinsic signaling (key intracellular cascades, metabolism, neurotransmitters) [4,5], as well as extrinsic signals like stress, exercise, and pharmacological agents [6,7]. Together, these factors regulate the differentiation, maturation, and even fate determination of NSCs [8].

Propofol is one of the most widely used intravenous anesthetics [9]. Previous studies have reported that high doses of propofol administration during early postnatal life suppressed hippocampal neurogenesis and impaired cognitive function, while addiction is a main problem of repeated use [10,11,12,13]. In contrast, low-dose propofol may promote neurogenesis [10,14,15]. Moreover, other research has shown that sub-anesthetic doses of propofol can alleviate anxiety induced by chronic stress, pain, and so on [16,17]. Clinical studies have further demonstrated that sub-hypnotic doses of propofol can prevent postoperative nausea and vomiting, provide analgesic effects, and improve mood in patients with depression [18,19,20]. These studies collectively support potential dose-dependent effects of propofol on the nervous system, though single-cell evidence on neurogenic fate regulation remains scarce.

In our previous work, we administered 4 mg/kg of propofol (a reported sub-anesthetic dose [21]) via intraperitoneal injection to PND7 C57BL/6N mice. Plasma concentrations in the L-Propofol group were measured and confirmed to fall within clinically relevant sedative levels [22], supporting the translational relevance of our experimental design. We found enhanced learning abilities and overall increased developing cell proportions after low-dose propofol treatment, revealed by single-nuclei sequencing [21]. However, due to limited analytical capabilities at the time, we did not delve deeply into the sequencing data, leaving the differentiation lineages and expression profile changes in the hippocampus after low-dose propofol treatment unclear.

Beyond these dose-dependent neuroprotective or neurotoxic effects, elucidating the molecular mechanisms—particularly, how propofol regulates gene expression and transcriptional networks—is crucial for understanding its impact on hippocampal neurogenesis. Emerging evidence shows that propofol can modulate intracellular signaling and gene expression beyond its anesthetic effects [23], such as PI3K/AKT signaling [24], mitochondrial modulation [25], and epigenetic mechanisms [26]. These regulatory effects highlight that propofol-induced transcriptional modulation should be considered when evaluating its impact on neurogenesis.

It is generally believed that the final fate of postnatal hippocampal neurogenesis is the generation of granule cells (GRCs). Hippocampal NSCs originate from a population of self-renewing, multipotent radial glia-like (RGL) cells, which maintain stemness while producing neural intermediate progenitor cells (nIPCs). Then, nIPCs undergo rapid, extensive proliferation and gradually differentiate into neuroblasts (NB1 and NB2), immature granule cells (imGCs), and eventually mature granule cells (mGCs), contributing to the formation of learning circuits [27]. In our previous work, we did not successfully conduct detailed clustering and expression profiling of the various stages of imGCs following low-dose propofol treatment.

Another key cell type involved in hippocampal function is the pyramidal cells, primarily located in the CA3 and CA1 regions. Developmental studies indicate that pyramidal neurons in the mouse hippocampus are generated between embryo day 12 (E12) and embryo day 18 (E18) [28,29], showing significant spatial and temporal developmental differences compared to dentate granule cells [28]. Recent research using nonspecific labeling techniques clarified that pyramidal neurons arise exclusively from embryonic neurogenesis, with no postnatal replenishment [30]. Meanwhile, in a single-cell transcriptomic study on hippocampal developmental origins, a population of immature pyramidal cells is present exclusively during the perinatal period (embryo day 16 (E16)–postnatal day 5 (PND5)) but absent in juvenile and adult hippocampi [31]. This suggests that both granule and pyramidal neuron maturation may occur postnatally in the hippocampal DG. It remains to be determined whether these two maturation trajectories are present in our previous hippocampal single-cell sequencing data. Furthermore, it is still unclear how low-dose propofol affects specific neurogenic patterns and whether it regulates neuronal maturation. These questions warrant further investigation.

To decipher the molecular mechanisms underlying low-dose propofol’s effects on hippocampal neurogenesis, we conducted a comprehensive bioinformatics reanalysis of our previously published single-nucleus RNA-sequencing (snRNA-seq) dataset. We refined cell clustering across different stages of neuronal differentiation and maturation in the hippocampus and elucidated cell-type-specific transcriptional alterations induced by low-dose propofol. By integrating advanced computational approaches—including trajectory inference, pathway enrichment, and cell–cell communication analysis—we revealed detailed neurogenic maturation patterns and molecular lineage trajectories following low-dose propofol administration. This analysis extends our earlier work, which primarily described overall changes in developing cell proportions, and provides new mechanistic insights into neuronal maturation shifts by propofol.

## 2. Methods

### 2.1. snRNA-Seq Data Processing

This study utilized previously generated snRNA-seq data from the mouse hippocampus treated with low-dose propofol (Diprivan, AstraZeneca, Cambridge, England). A total of 22,457 (11,347 in Control and 11,110 in L-Propofol) nuclei were isolated. Each sample pooled hippocampi from 5–6 PND10 pups, and a 10× Genomics platform was used for single-nuclei transcriptomes. The raw data were deposited in the Gene Expression Omnibus (GEO) database under accession number GSE186216.

Data processing was conducted using the OmicStudio platform developed by LC Sciences (Hangzhou, China). Raw sequencing data were demultiplexed and converted to FASTQ format using Illumina (San Diego, CA, USA) bcl2fastq software (version 2.20). Subsequent preprocessing, including quality control, barcode processing, genome alignment, and transcript quantification, was performed using the Cell Ranger software suite (version 6.1.1, 10× Genomics, Pleasanton, CA, USA).

The gene expression matrices generated by Cell Ranger were analyzed in R (version 4.1.2) using the Seurat package (version 4.1.1) to associate barcodes with cell identifiers and unique molecular identifiers (UMIs). Cells with mitochondrial UMI counts greater than 25% or fewer than 500 detected genes were considered low-quality and removed. Doublets were identified and excluded using DoubletFinder (version 2.0.3). After removing low-quality cells, 20,530 cells (10,363 in Control, 10,167 in Propofol) passed the quality control threshold, with a medium of approximately 1900 detected genes per nucleus. Then, the gene expression matrix was normalized using Seurat’s standard normalization function, and the top 2000 highly variable features were identified for downstream analysis.

Principal component analysis (PCA) was performed using the RunPCA()function, and the top 20 principal components were selected for downstream clustering. Clustering was conducted using the FindNeighbors() and FindClusters() functions, with the resolution parameter set to 0.8. Dimensionality reduction and visualization were carried out using the RunUMAP() function.

### 2.2. Pseudotime Analysis

Nine identified cell subpopulations were extracted from the dataset to infer differentiation trajectories. The Seurat object was converted into a CellDataSet object and analyzed using the Monocle2 package (version 2.22.0, Trapnell Lab, Department of Genome Sciences, University of Washington, Seattle, WA, USA). Dimensionality reduction was performed using the reduceDimension() function, followed by cell ordering along pseudotime using the orderCells() function to identify key branching points.

Branch-dependent genes were identified using the Branch Expression Analysis Modeling (BEAM) method in Monocle2, with a significance threshold of *p* < 0.05. The expression patterns of significant genes along branches were visualized using the plot_genes_branched_heatmap() function.

### 2.3. Differentially Expressed Gene (DEG) Identification

Differential expression analysis was conducted using the FindMarkers() function in Seurat (version 4.1.1), employing the bimod statistical test. Genes with a log_2_ fold change (Log_2_FC) ≥ 0.26 and an adjusted *p*-value (Bonferroni correction) < 0.01 were considered significantly differentially expressed. Identified DEGs were subjected to further biological function and pathway enrichment analyses.

### 2.4. Cell–Cell Communication Analysis

Cell–cell communication was analyzed using the CellChat package (version 2.1.2), based on its built-in mouse ligand–receptor interaction database. Annotated Seurat objects were split into Control and L-Propofol-treated groups using the SplitObject() function, and communication networks were constructed for each group separately.

Using the standard CellChat workflow, the number and strength of intercellular interactions within each group were assessed. The two networks were then merged using the mergeCellChat() function to compare communication patterns between groups. Particular attention was given to identifying significant ligand–receptor interactions involving neural progenitor cells (NPC) and other cell types. Pathway enrichment was evaluated via hypergeometric testing to determine statistically enriched signaling interactions.

## 3. Results

### 3.1. Cell Type Annotation of Single-Nucleus Transcriptomic Landscape in Neonatal Mouse Hippocampus

**Figure 1:**
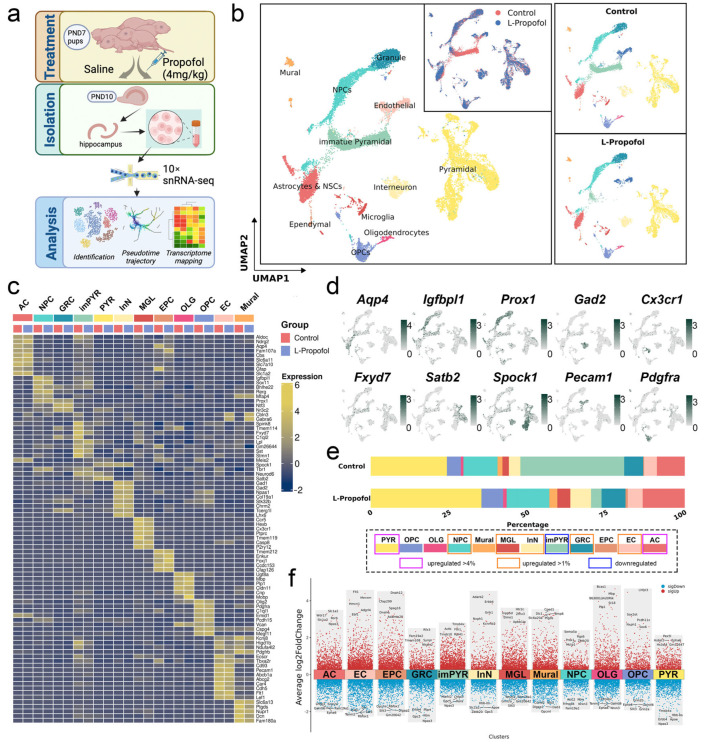
Single-nucleus transcriptomic profiling reveals propofol-induced shifts in hippocampal cell populations: (**a**) Schematic of hippocampal sample collection and processing. (**b**) UMAP embedding of all hippocampal nuclei, colored by cluster annotation (**left**). Integration plot of cells from both Control and L-Propofol groups (**top center**), with separate UMAPs showing the distribution of annotated cell types in the Control group (**top right**) and in the L-Propofol group (**bottom right**). (**c**) Heatmap of the mean expression levels of marker genes defining the 12 annotated cell types under each treatment condition. (**d**) UMAP feature plots for key lineage-specific markers; high expression is shown in green and low expression in gray. (**e**) Bar graph depicting proportional changes in each cell population following L-Propofol treatment. (**f**) Volcano plot of differentially expressed genes across all subpopulations, with upregulated genes in red and downregulated genes in blue; the top five most significant DEGs are labeled. Annotations: AC (astrocytes & neural stem cells), NPC (neural progenitor cells), GRC (granulocytes), imPYR (immature pyramidal cells), PYR (pyramidal cells), InN (interneurons), MGL (microglia), EPC (ependymocytes), OLG (oligodendrocytes), OPC (oligodendrocyte precursor cells), EC (endothelial cells), and Mural (mural cells).

In this study, we analyzed a previously established snRNA-seq dataset comprising hippocampal tissue samples from the Control and L-Propofol groups. Strict quality control was applied following criteria consistent with our previous study [21], and the tissue sampling protocol was illustrated in Figure 1a, which outlined the experimental design and hippocampal dissection workflow. Using uniform manifold approximation and projection (UMAP) for dimensionality reduction and clustering, we annotated cell types by integrating previously reported DEGs and canonical markers [31,32] (Figure 1b). We identified twelve major cell types in total. Astrocytes & neural stem cells (AC) were defined by *Aqp4* expression, while NPC were marked by *Igfbpl1*. GRC expressed *Prox1*, and immature pyramidal neurons (imPYR) were characterized by FXYD domain-containing ion transport regulator (*Fxyd7*). Other identified types included mature pyramidal neurons (PYR; *Satb2*, *Spock1*), interneurons (InN; *Gad2*), microglia (MGL; *Cx3cr1*), ependymocytes (EPC; *Trem212*, *Enkur*), oligodendrocytes (OLG; *Mbp*, *Ugt8a*, *Plp1*), oligodendrocyte precursor cells (OPC; *Pdgfra*), endothelial cells (EC; *Pecam1*), and mural cells (Mural; *Slc6a13*, *Pdgfrb*). Expression analysis of these marker genes showed high consistency across groups (Figure 1c); representative marker expression profiles are presented in Figure 1d.

Importantly, we identified a distinct cell population that showed low expression of most lineage-specific markers but relatively strongly expressed *Fxyd7*—a previously reported feature gene of immature neurons [31,33]. This cluster also expressed pyramidal neuron markers *Spink8*, *Tmem114*, *Lpl*, and *Stmn1*, but lacked mature markers like *Meis2* and *Spock1*. These features collectively support its designation as imPYR.

To further characterize potential cellular remodeling induced by L-Propofol, we quantitatively compared hippocampal cell subtypes between groups and observed striking differences (Figure 1b,e). In the L-Propofol group, the fraction of imPYR sharply declined from 33.0% to 3.3% (approximately 10-fold reduction), representing the most pronounced change. In contrast, the proportions of PYR and AC increased markedly by over 4% (from 24.3% to 35.3% and from 8.9% to 13.4%, respectively). The GRC also showed a moderate expansion (6.1% to 7.6%), consistent with enhanced neurogenesis. Additionally, NPCs, MGL, InN, and EC exhibited smaller increases (by over 1%).

These findings suggest that low-dose propofol reduces imPYR abundance while promoting the expansion of other cell types, particularly AC and PYR. These quantitative observations complement the qualitative annotations and highlight pronounced lineage shifts, especially within the pyramidal and granule neuron populations following L-Propofol exposure. Furthermore, differential expression analysis across all cell types (Figure 1f) validated the distinct transcriptional identities of these clusters.

### 3.2. Low-Dose Propofol Alters the Direction of Neurogenic Lineage Differentiation

**Figure 2:**
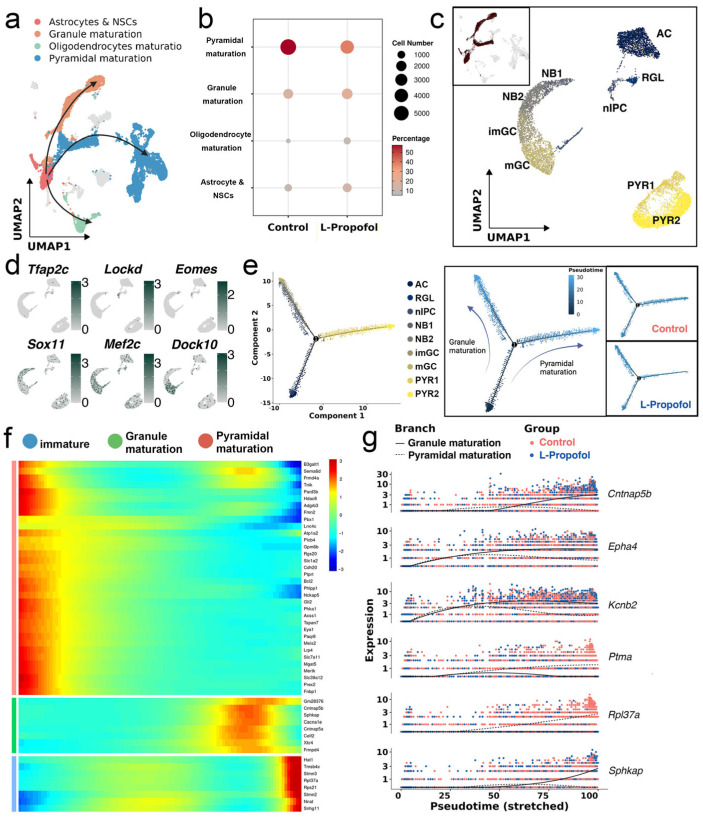
Low-dose propofol redirects hippocampal neurogenic trajectories: (**a**) UMAP embedding of 4 neurogenesis-associated differentiation directions, colored by cluster annotation. (**b**) Dot plot showing the proportion of each neurogenic subpopulation in Control and L-Propofol groups; dot size reflects relative abundance. (**c**) UMAP embedding of the re-clustered neurogenic cells, with 9 refined subclusters color-coded by annotation. (**d**) UMAP feature plots for representative differentially expressed genes across the subclusters; green indicates high expression and gray low expression. (**e**) Monocle2 trajectory analysis of the 9 subclusters: combined trajectory colored by cluster annotation (**left**), pseudotime progression from dark (origin) to light (terminal) (**middle**), and separate trajectories for Control (**top right**) and L-Propofol (**bottom right**) groups. (**f**) Heatmap of the top 50 branch-dependent genes identified by BEAM, illustrating transcriptional bifurcation at the lineage branch point. (**g**) Scatter plot of the top six branch-specific differential genes, showing their expression dynamics along the pseudotime axis. Annotations: AC (astrocytes), RGL (radial glia-like cells), nIPC (neural intermediate progenitor cells), NB1 (neuroblasts 1), NB2 (neuroblasts 2), imGC (immature granulocytes), mGC (mature granulocytes), PYR1 (pyramidal cell 1), and PYR2 (pyramidal cell 2).

Based on the defined cell populations above, we categorized the cells into three major differentiation trajectories: granule maturation, oligodendrocyte maturation, and pyramidal maturation (Figure 2a). In the L-Propofol group, pyramidal-lineage cells decreased significantly, whereas granule and oligodendrocyte lineages increased (Figure 2b). Among these changes, the sharp decline in imPYR abundance represented the most prominent shift.

To investigate potential redirection of neurogenesis, we analyzed relatively immature neuronal populations (AC, NPC, GRC, and imPYR) through refined subclustering. These nine subtypes identified represented the postnatal hippocampal neurogenesis continuum (Figure 2c,d). Among them, AC (expressing *Aqp4*) and RGL (marked by *Tfap2c*) correspond to quiescent NSCs that are developmentally related and thus not clearly separated in initial clustering. NPCs were further divided into nIPC (expressing *Lockd*) and two neuroblast populations: NB1 (expressing *Eomes*) and NB2 (expressing *Sox11*). The granule lineage included immature and mature granule cells—imGC (marked by *Mef2c*) and mGC (expressing *Dock10*). Pyramidal neurons were separated into PYR1 (marked by *Neurod6* and *Ntng1*) and PYR2 (marked by *Fxyd7*). These subpopulations represent immature stages of pyramidal neuron development and, for clarity, are collectively referred to here as pyramidal neurons.

Pseudotime trajectory analysis (Monocle2) revealed two main differentiation routes (Figure 2e). One toward mature granule neuron, progressing from RGL to nIPC to NB1, NB2 to imGC and mGC; and the other toward pyramidal neuron, with RGL to nIPC to a minority of NB1 to PYR1 and PYR2. In the Control group, cells were distributed relatively evenly along both trajectories. However, in the L-Propofol group, cells predominantly followed the granule lineage path, suggesting a shift in progenitor fate toward granule maturation after neonatal low-dose propofol administration.

To further explore this lineage bias, we applied BEAM, identifying the top 50 genes with significant branch-dependent expression (Figure 2f). Among these, *Cntnap5b*, *Epha4*, *Kcnb2*, and *Sphkap* were notably enriched in the granule lineage and exhibited a trajectory-specific expression pattern highly synchronized with the branch point (Figure 2g).

### 3.3. Propofol Cooperatively Regulates Neuronal Differentiation by Activating Synaptic Plasticity

**Figure 3:**
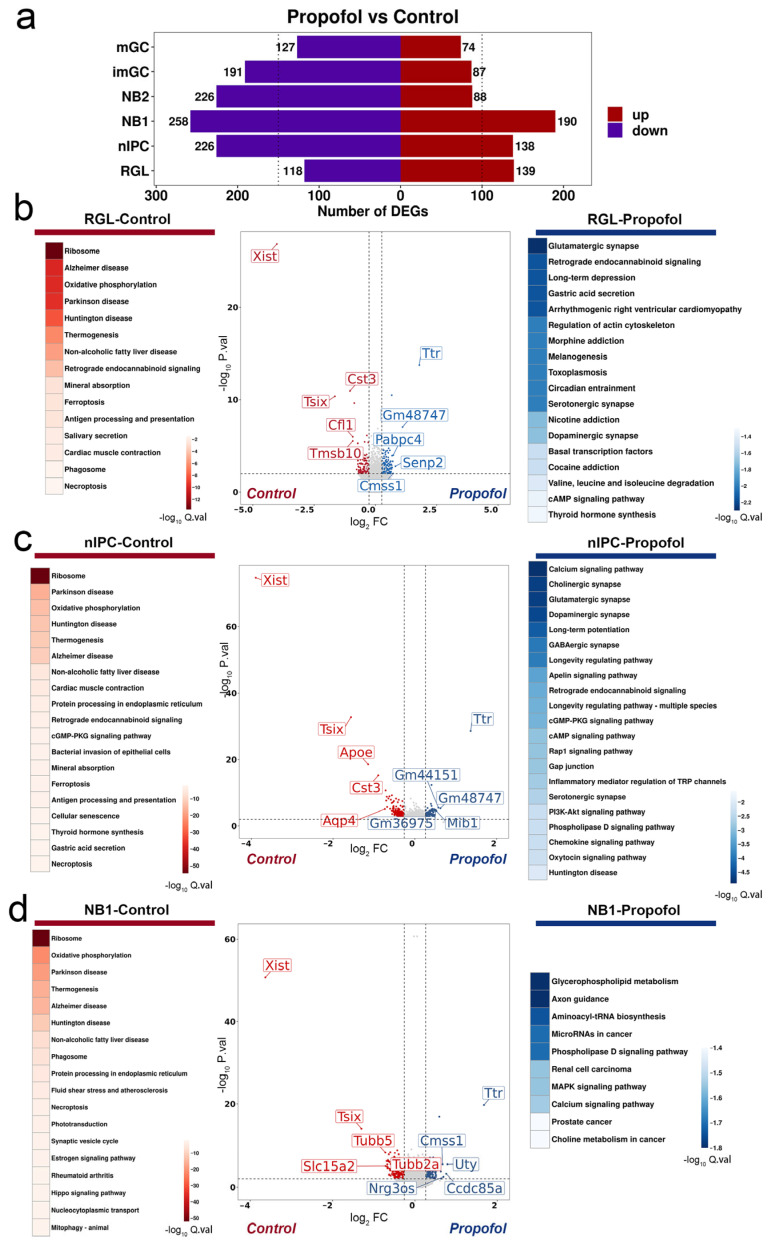
Low-dose propofol regulates synaptic plasticity pathways in the early granule maturation lineage: (**a**) Bar chart of the number of DEGs in all granule-lineage subclusters (RGL, nIPC, NB1, NB2, imGC, and mGC) after low-dose Propofol treatment. The left and right dashed lines indicate reference thresholds of 150 and 100 DEGs, respectively. (**b**–**d**) Volcano dot plots and significantly different GO enrichment categories for DEGs enriched in Control (red) and L-Propofol (blue) groups in early progenitors (RGL in (**b**), nIPC in (**c**), NB1 in (**d**)) after low-dose Propofol treatment. In the volcano plots (**b**–**d**), the vertical dashed lines indicate the log_2_ fold change threshold (|Log_2_FC| ≥ 0.26), and the horizontal dashed line indicates the adjusted *p*-value cutoff (Bonferroni-corrected *p* < 0.01). Annotations: DEG (differentially expressed gene), RGL (radial glia-like cells), nIPC (neural intermediate progenitor cells), NB1 (neuroblasts 1), imGC (immature granulocytes), mGC (mature granulocytes), GO (Gene Ontology), L-Propofol (low-dose propofol).

Within the granule cell lineage, we identified DEGs between L-Propofol (low-dose propofol) and Control groups using thresholds of |log_2_FC| ≥ 0.26 and adjusted *p* < 0.05. Aside from RGL, all later subpopulations (nIPC, NB1, NB2, imGC, and mGC) exhibited more downregulated than upregulated genes. The total number of DEGs decreased progressively along the differentiation trajectory (Figure 3a), indicating that low-dose propofol exerts the strongest transcriptional effect on early-stage progenitors.

Gene Ontology (GO) enrichment of Control-enriched DEGs revealed significant enrichment in “cellular component” categories—such as ribosome biogenesis, mitochondrial oxidative phosphorylation, endoplasmic reticulum protein processing, and synaptic vesicle cycling—as shown in early progenitor populations (RGL, nIPC, and NB1) in Figure 3b–d and other populations (NB2, imGC, and mGC) in Figure S1. These results suggest that low-dose propofol alters the subcellular localization of basic metabolic processes.

In contrast, upregulated genes in the L-Propofol group showed minimal enrichment in late-stage cells (imGC, mGC). Early progenitors (nIPC, NB1, NB2) were specifically enriched for neurodevelopmental and synaptic remodeling pathways, including long-term potentiation (LTP), axon guidance, and memory-related processes, with nIPC displaying the most selective activation. Notably, nIPC also uniquely upregulated GABAergic, glutamatergic, and cholinergic synapse pathways, implying enhanced synaptic homeostasis and circuit reorganization capacity at this stage, as reflected by the enriched synaptic signaling pathways in Figure 3c. Together, these data suggested that low-dose propofol shapes neuronal fate through stage-specific modulation of metabolic and synaptic plasticity programs, a process we term “Immaturity-Dependent.”

### 3.4. Cell–Cell Communication Analysis Highlights the Nrxn Signaling Axis

**Figure 4:**
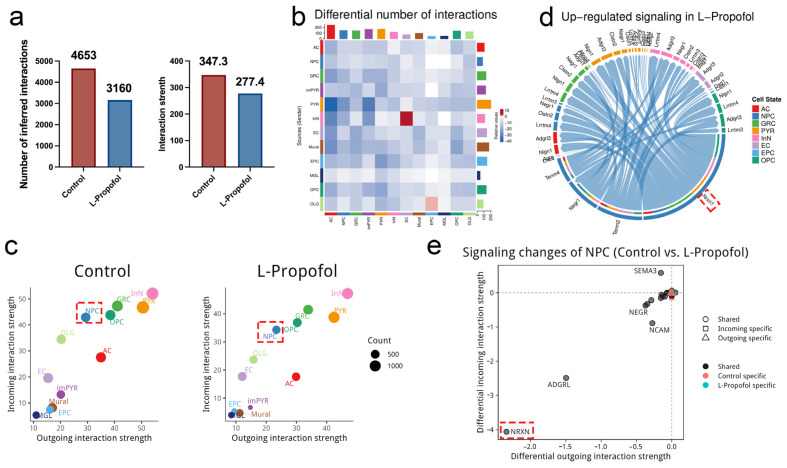
CellChat analysis reveals *Nrxn* signaling dominance in NPC following L-propofol treatment: (**a**) Bar plots quantify interaction numbers and communication strength across cell populations in the Control versus L-propofol groups. (**b**) Heatmap displays interpopulation interaction counts, revealing a marked reduction in total cellular communication events following L-propofol treatment. Red/blue gradients denote signaling enrichment in Control/L-propofol groups, respectively. Cell subtypes are color-coded as indicated. (**c**) Communication role analysis identifies NPC as predominant signal receivers across all lineages after low-dose propofol exposure, visualized through source-target probability matrices. (**d**) Circos plot visualizes significantly upregulated ligand-receptor pairs between NPC and all populations in the L-Propofol group, with lower/upper semicircles representing ligands/receptors. Ribbon width correlates with interaction strength. The red dashed box highlights the Nrxn1 signaling pathway. (**e**) Differential signaling analysis shows enhanced outgoing and incoming interactions in Nrxn pathways in NPC after low-dose propofol exposure. Circle indicates conserved pathways; square marks incoming-specific signals; triangles mark outgoing-specific signals. Black/red/green colors denote share/Control/L-Propofol-specific signals. Annotations: Nrxn (Neurexin), NPC (neural progenitor cells), L-Propofol (low-dose propofol).

To mechanistically dissect the propofol-induced neurogenic shift, we applied CellChat to infer ligand–receptor interactions. In the hippocampus, secreted signaling dominated the communication network. After L-Propofol treatment, this network was markedly remodeled, as most cell types exhibited reduced numbers of both outgoing and incoming signals (Figure 4a,b). Further analysis revealed a global reduction in cell–cell communication strength following low-dose propofol treatment (Figure 4a,c). In particular, NPC showed significant functional changes, experiencing an overall decrease in signal strength, as illustrated in Figure 4c. Based on our previous transcriptomic evidence [13] and the known central role of NPC in hippocampal development, we conducted a focused analysis of their enhanced interlineage interactions. We found that Neurexin1 (Nrxn1) signaling was significantly enhanced and formed extensive cross-lineage ligand–receptor connections, as visualized in Figure 4d.

Nrxn1, a member of the Neurexin (Nrxn) family of presynaptic adhesion molecules, undergoes complex alternative splicing to establish diverse neural circuits and has been implicated in neuropsychiatric disorders [34,35]. Further analysis of NPC–NPC interactions revealed that both incoming and outgoing Nrxn signals were significantly increased following low-dose propofol treatment, quantified in Figure 4e. These results indicate that Nrxn pathway activation in NPC is a critical driver of progenitor fate redirection. Collectively, these data demonstrate that low-dose propofol promotes granule neuron differentiation by upregulating Nrxn signaling in NPC.

## 4. Discussion

The effects of anesthetics on the development of the immature brain remain incompletely characterized. In this study, we utilized a previously established snRNA-seq dataset to perform reclustering and redefinition of hippocampal cells in PND10 mice following low-dose propofol treatment. For the first time, we identified a population of “immature pyramidal neurons” in the hippocampus at this developmental stage. Our findings suggest that propofol may redirect neurogenic differentiation from the pyramidal lineage toward granule maturation. After defining six granule neuron maturation-associated subpopulations, we further analyzed transcriptional differences between groups. We found that propofol primarily affects nIPC, which exhibits the highest stemness. At this stage, propofol preferentially activates neurogenic programs that promote neuronal maturation. Moreover, we identified Nrxn signaling as a key mediator associated with NPC activation. Collectively, these findings indicate that low-dose propofol not only redirects hippocampal neurogenic trajectories during early postnatal development but also exerts its cognitive-enhancing effects primarily by stimulating NPC at their highest stemness state.

Postnatally, the hippocampal DG undergoes continuous neurogenesis, which plays a crucial role in hippocampus-related neural functions. It is widely accepted that postnatal hippocampal neurogenesis primarily contributes to the generation of granule neurons that replenish the DG granule cell layer and directly participate in the formation of hippocampal learning circuits [27]. In contrast, pyramidal neurons in the CA1 and CA3 regions are thought to complete their differentiation during prenatal development (mid-to-late gestation), and there is currently no direct evidence that they benefit from postnatal neurogenesis [28]. By referring to recent single-cell transcriptomic studies of hippocampal development, we identified a population of imPYR marked by *Ntng1*, *Fxyd7*, and *Pcp4* [31,33,36,37], which, apart from previous reports in PND5 mice [31], had not been clearly defined in the postnatal hippocampus. This identification suggests that, in early postnatal life, hippocampal neurogenesis may involve not only the canonical granule cell trajectory but also a minor pyramidal differentiation pathway.

We then compared the proportions of neurogenic subpopulations between the Control and L-Propofol groups. The results revealed that propofol-treated hippocampi exhibited an increased proportion of granule lineage-related cells and a marked decrease in imPYR populations. Two possible interpretations could explain this phenomenon. First, propofol might redirect progenitor cells originally destined for pyramidal differentiation toward the granule cell lineage. Second, it might accelerate imPYR maturation and migration. Although our current data cannot fully distinguish between these possibilities, trajectory analysis revealed two differentiation directions—toward granule cells and toward pyramidal neurons—with a notable reduction in pyramidal trajectory cells in the propofol-treated group. This supports the hypothesis that propofol redirects progenitor fate away from pyramidal and toward granule neuron differentiation. While we cannot exclude the possibility of accelerated pyramidal maturation, the well-established importance of granule neurogenesis for hippocampal function [1,27,38,39] suggests that the observed enhancement of granule neurogenesis following propofol treatment may play a decisive role in long-term cognitive improvement.

Since the discovery of ongoing neurogenesis in the postnatal brain in the late 20th century [40], the stages of granule neuron development have been extensively characterized [41]. The neurogenic progression from neural stem cells involves three phases: proliferation, differentiation, and maturation. During the differentiation phase, cells are further classified by their degree of stemness into nIPC and two neuroblast stages—NB1 and NB2 [41]. In this study, we successfully delineated six granule neurogenesis-associated subtypes based on single-cell transcriptomic profiles, consistent with previous reports [31,42], thereby validating our subtype classification and supporting a refined understanding of granule neuron development following propofol treatment.

Further GO analysis of downregulated genes demonstrated predominant enrichment in cellular components, particularly subcellular localizations, with minimal variation across cell types. In contrast, upregulated genes were primarily enriched in biological processes and molecular functions, with significant differences concentrated in NPC, especially nIPC. These findings collectively reveal three key features of transcriptional regulation in granule lineage following low-dose propofol exposure: (1) a shift in cellular localization of biological responses across all neurogenic subtypes—from cytoplasmic/nuclear activity to membrane-associated activity (plasma membrane, nuclear envelope, organelle membranes), possibly reflecting the membrane-targeting effects of the highly lipophilic propofol molecule; (2) a prominent effect on neural progenitor and neuroblast populations, particularly nIPC, highlighting their heightened sensitivity to propofol; and (3) activation of neurogenic maturation pathways in progenitor populations, including synaptogenesis, synaptic transmission, dendrite morphogenesis, and memory-related processes. Although *Xist* expression was detected in Figure 3d, all animals used in this study were male, suggesting that this signal is unlikely to reflect sex-related regulation and is more likely due to technical noise inherent to single-nucleus RNA-seq analysis. These specific mechanisms have not been reported in previous studies of anesthetic-induced effects on neurodevelopment.

To link these intracellular shifts with changes in the hippocampal niche, we conducted systematic mapping of intercellular communication networks using CellChat. We found that low-dose propofol selectively enhances Nrxn signaling in NPC. As presynaptic cell-adhesion molecules, Neurexins are essential for synapse formation and neurotransmitter regulation, and their interaction with Neuroligin-1 (NL1) can counteract glutamate-induced synaptic inhibition to stabilize synapses [43]. Nrxn also undergoes complex alternative splicing to establish diverse neural circuits and has been implicated in neuropsychiatric disorders [34,35]. Dysregulation of Nrxn is associated with synaptic dysfunction and neurodevelopmental disorders such as autism spectrum disorder [44] and schizophrenia [45], underscoring its critical role in synaptic formation and network balance. Moreover, research found that Neurexin–Neuroligin interaction also contributes to LTP and memory consolidation in the hippocampus [46,47], further supporting its potential involvement in propofol-induced modulation of synaptic and cognitive functions.

Importantly, the upregulation of synaptic stability and memory-related gene programs revealed by GO analysis is consistent with the enhancement of Nrxn signaling observed here, suggesting that low-dose propofol may reinforce synaptic connectivity and memory-related processes through Nrxn-mediated pathways.

Taken together, these results indicate that enhanced Nrxn signaling in NPC may act as a pivotal mediator linking transcriptional activation of synaptic genes to functional remodeling of neuronal circuits—facilitating membrane reorganization, synaptic maturation, and ultimately promoting the redirection of progenitors toward a granule neuron fate under low-dose propofol exposure.

Although direct mechanistic links between neonatal propofol exposure and Nrxn regulation await further validation, the convergence of transcriptional, subcellular, and intercellular remodeling delineated here offers a distinctive contribution to the field of anesthetic neurodevelopmental research.

Despite providing novel insights, our study has several key limitations. Firstly, this study focused on a single developmental stage (PND7) and one propofol dose, precluding examination of dose-relevant or age-dependent effects. This is an important limitation, as neuronal vulnerability and synaptic maturation vary substantially across developmental periods. The timing of anesthetic exposure may exert distinct neurotoxic or neuroprotective effects depending on brain maturity. Therefore, findings obtained at PND7 may not fully reflect responses that occur at earlier or later postnatal stages. It should also be noted that the snRNA-seq analysis was performed using one biological replicate per group. Although each sample included hippocampi from multiple pups, this limitation may restrict the generalizability of the findings and warrants further validation in larger cohorts. Moreover, the observed phenomena are limited to the snRNA-seq captures, which include only nuclear transcripts and omit cytoplasmic RNAs, and they lack direct in vivo validation, warranting further investigation.

## 5. Conclusions

Above all, these findings expand our understanding of how low-dose propofol modulates early postnatal brain development and underscore the importance of progenitor-specific and synapse-oriented mechanisms in mediating cognitive outcomes. Future studies should incorporate multiple developmental stages, varied dosage, and direct perturbations of Nrxn pathways, coupled with electrophysiological and behavioral assessments, to better delineate how propofol affects neurodevelopmental trajectories and whether the observed molecular and cellular alterations are stage- or dose-specific. Such approaches will be essential to validate these insights and translate them into potential cognitive-enhancement strategies.

## Data Availability

The datasets analyzed during the current study are available in the GEO database under accession number GSE186216.

## Supplementary Materials

The following supporting information can be downloaded at: https://www.mdpi.com/article/10.3390/biomedicines13112806/s1. Figure S1. Low-dose propofol regulates synaptic plasticity pathways along the granule maturation lineage: (a–c) Volcano dot plots and significantly different GO enrichment categories for DEGs enriched in Control (red) and L-Propofol (blue) groups in late granule lineage (NB2 in (a), imGC in (b), mGC in (c)) after L-Propofol treatment. In the volcano plots (a–c), the vertical dashed lines indicate the log_2_ fold change threshold (|Log_2_FC| ≥ 0.26), and the horizontal dashed line indicates the adjusted *p*-value cutoff (Bonferroni-corrected *p* < 0.01).

## Abbreviations

AC	Astrocyte & neural stem cells
BEAM	Branch Expression Analysis Modeling
DEG	Differentially expressed gene
DG	Dentate gyrus
E12	Embryo day 12
EC	Endothelial cells
EPC	Ependymocytes
GEO	Gene Expression Omnibus
GO	Gene Ontology
GRC	Granule cells
imGC	Immature granule cells
imPYR	Immature pyramidal neurons
InN	Interneurons
L-Propofo	low-dose propofol
MGL	Microglia
mGC	Mature granule cells
Mural	Mural cells
NB	Neuroblasts
NL1	Neuroligin-1
Nrxn	Neurexin
nIPC	Neural intermediate progenitor cells
NPC	Neural progenitor cells
NSCs	Neural stem cells
OLG	Oligodendrocytes
OPC	Oligodendrocyte precursor cells
PCA	Principal component analysis
PND	Postnatal day
PYR	Mature pyramidal neurons
RGL	Radial glia-like cells
snRNA-seq	Single-nucleus RNA-sequencing
UMAP	Uniform Manifold Approximation and Projection

## References

[1] Kuhn H.G., Toda T., Gage F.H. (2018). Adult Hippocampal Neurogenesis: A Coming-of-Age Story. J. Neurosci..

[2] Franjic D., Skarica M., Ma S., Arellano J.I., Tebbenkamp A.T.N., Choi J., Xu C., Li Q., Morozov Y.M., Andrijevic D. (2022). Transcriptomic Taxonomy and Neurogenic Trajectories of Adult Human, Macaque, and Pig Hippocampal and Entorhinal Cells. Neuron.

[3] Kjell J., Fischer-Sternjak J., Thompson A.J., Friess C., Sticco M.J., Salinas F., Cox J., Martinelli D.C., Ninkovic J., Franze K. (2020). Defining the Adult Neural Stem Cell Niche Proteome Identifies Key Regulators of Adult Neurogenesis. Cell Stem Cell.

[4] Cope E.C., Gould E. (2019). Adult Neurogenesis, Glia, and the Extracellular Matrix. Cell Stem Cell.

[5] Zhao Y., Liu H., Zhang Q., Zhang Y. (2020). The Functions of Long Non-Coding RNAs in Neural Stem Cell Proliferation and Differentiation. Cell Biosci..

[6] Horowitz A.M., Fan X., Bieri G., Smith L.K., Sanchez-Diaz C.I., Schroer A.B., Gontier G., Casaletto K.B., Kramer J.H., Williams K.E. (2020). Blood Factors Transfer Beneficial Effects of Exercise on Neurogenesis and Cognition to the Aged Brain. Science.

[7] Jung S., Choe S., Woo H., Jeong H., An H.-K., Moon H., Ryu H.Y., Yeo B.K., Lee Y.W., Choi H. (2020). Autophagic Death of Neural Stem Cells Mediates Chronic Stress-Induced Decline of Adult Hippocampal Neurogenesis and Cognitive Deficits. Autophagy.

[8] Gonçalves J.T., Schafer S.T., Gage F.H. (2016). Adult Neurogenesis in the Hippocampus: From Stem Cells to Behavior. Cell.

[9] Kim K.-M., Choi B.-M., Park S.-W., Lee S.-H., Christensen L.V., Zhou J., Yoo B.-H., Shin H.-W., Bae K.-S., Kern S.E. (2007). Pharmacokinetics and Pharmacodynamics of Propofol Microemulsion and Lipid Emulsion after an Intravenous Bolus and Variable Rate Infusion. Anesthesiology.

[10] Chang C., Bai W., Li J., Huo S., Wang T., Shao J. (2023). Effects of Subchronic Propofol Administration on the Proliferation and Differentiation of Neural Stem Cells in Rat Hippocampus. Curr. Ther. Res. Clin. Exp..

[11] Huang J., Jing S., Chen X., Bao X., Du Z., Li H., Yang T., Fan X. (2016). Propofol Administration during Early Postnatal Life Suppresses Hippocampal Neurogenesis. Mol. Neurobiol..

[12] Kim J.L., Bulthuis N.E., Cameron H.A. (2020). The Effects of Anesthesia on Adult Hippocampal Neurogenesis. Front. Neurosci..

[13] Yang M., Zhang Y. (2024). Propofol Addiction: The Mechanism Issues We Need to Know. Anesthesiol. Perioper. Sci..

[14] Qiao H., Li Y., Xu Z., Li W., Fu Z., Wang Y., King A., Wei H. (2017). Propofol Affects Neurodegeneration and Neurogenesis by Regulation of Autophagy via Effects on Intracellular Calcium Homeostasis. Anesthesiology.

[15] Wei W., Zhang F., Chen H., Tang Y., Xing T., Luo Q., Yu L., Du J., Shen J., Zhang L. (2018). Toxoplasma Gondii Dense Granule Protein 15 Induces Apoptosis in Choriocarcinoma JEG-3 Cells through Endoplasmic Reticulum Stress. Parasit. Vectors.

[16] Jiang S., Ge D., Song B., Deng X., Liu Z., He J., Sun J., Zhu Z., Meng Z., Zhu Y. (2025). Subanesthetic Propofol Alleviates Chronic Stress-Induced Anxiety by Enhancing VTADA Neurons’ Activity. Neuropharmacology.

[17] Yu L., Zhu X., Peng K., Qin H., Yang K., Cai F., Hu J., Zhang Y. (2024). Propofol Alleviates Anxiety-Like Behaviors Associated with Pain by Inhibiting the Hyperactivity of PVNCRH Neurons via GABAA Receptor Β3 Subunits. Adv. Sci..

[18] Zacny J.P., Coalson D.W., Young C.J., Klafta J.M., Lichtor J.L., Rupani G., Thapar P., Apfelbaum J.L. (1996). Propofol at Conscious Sedation Doses Produces Mild Analgesia to Cold Pressor-Induced Pain in Healthy Volunteers. J. Clin. Anesth..

[19] Montgomery J.E., Sutherland C.J., Kestin I.G., Sneyd J.R. (1996). Infusions of Subhypnotic Doses of Propofol for the Prevention of Postoperative Nausea and Vomiting. Anaesthesia.

[20] Feldman D.A., Jones K.G., Vonesh L.C., Jacobs R., Hoffman N., Lybbert C., Huang J., Kuck K., Odell D., Tadler S.C. (2025). Immediate Effects of Propofol on Mood: A Randomized Comparison of Two Doses in a Cohort with Depression. Psychopharmacology.

[21] Chen K., Lu D., Yang X., Zhou R., Lan L., Wu Y., Wang C., Xu X., Jiang M.H., Wei M. (2022). Enhanced Hippocampal Neurogenesis Mediated by PGC-1α-Activated OXPHOS after Neonatal Low-Dose Propofol Exposure. Front. Aging Neurosci..

[22] Kim I.T. (2007). Adjustable Strabismus Surgery under Intravenous Anesthesia with Propofol and Fentanyl. J. Korean Ophthalmol. Soc..

[23] Upton D.H., Popovic K., Fulton R., Kassiou M. (2020). Anaesthetic-Dependent Changes in Gene Expression Following Acute and Chronic Exposure in the Rodent Brain. Sci. Rep..

[24] Wang L., Tang X., Li S. (2022). Propofol Promotes Migration, Alleviates Inflammation, and Apoptosis of Lipopolysaccharide-Induced Human Pulmonary Microvascular Endothelial Cells by Activating PI3K/AKT Signaling Pathway via Upregulating APOM Expression. Drug Dev. Res..

[25] Zhong H., Song R., Pang Q., Liu Y., Zhuang J., Chen Y., Hu J., Hu J., Liu Y., Liu Z. (2018). Propofol Inhibits Parthanatos via ROS-ER-Calcium-Mitochondria Signal Pathway in Vivo and Vitro. Cell Death Dis..

[26] Tabnak P., Masrouri S., Geraylow K.R., Zarei M., Esmailpoor Z.H. (2021). Targeting MiRNAs with Anesthetics in Cancer: Current Understanding and Future Perspectives. Biomed. Pharmacother..

[27] Kozareva D.A., Cryan J.F., Nolan Y.M. (2019). Born This Way: Hippocampal Neurogenesis across the Lifespan. Aging Cell.

[28] Cavalieri D., Angelova A., Islah A., Lopez C., Bocchio M., Bollmann Y., Baude A., Cossart R. (2021). CA1 Pyramidal Cell Diversity Is Rooted in the Time of Neurogenesis. Elife.

[29] Subramanian L., Tole S. (2009). Mechanisms Underlying the Specification, Positional Regulation, and Function of the Cortical Hem. Cereb. Cortex.

[30] Bond A.M., Berg D.A., Lee S., Garcia-Epelboim A.S., Adusumilli V.S., Ming G., Song H. (2020). Differential Timing and Coordination of Neurogenesis and Astrogenesis in Developing Mouse Hippocampal Subregions. Brain Sci..

[31] Hochgerner H., Zeisel A., Lönnerberg P., Linnarsson S. (2018). Conserved Properties of Dentate Gyrus Neurogenesis across Postnatal Development Revealed by Single-Cell RNA Sequencing. Nat. Neurosci..

[32] Zeisel A., Muñoz-Manchado A.B., Codeluppi S., Lönnerberg P., La Manno G., Juréus A., Marques S., Munguba H., He L., Betsholtz C. (2015). Brain Structure. Cell Types in the Mouse Cortex and Hippocampus Revealed by Single-Cell RNA-Seq. Science.

[33] Yao J., Dai S., Zhu R., Tan J., Zhao Q., Yin Y., Sun J., Du X., Ge L., Xu J. (2024). Deciphering Molecular Heterogeneity and Dynamics of Human Hippocampal Neural Stem Cells at Different Ages and Injury States. Elife.

[34] Fernando M.B., Fan Y., Zhang Y., Tokolyi A., Murphy A.N., Kammourh S., Deans P.J.M., Ghorbani S., Onatzevitch R., Pero A. (2025). Phenotypic Complexities of Rare Heterozygous Neurexin-1 Deletions. Nature.

[35] Hu Z., Xiao X., Zhang Z., Li M. (2019). Genetic Insights and Neurobiological Implications from NRXN1 in Neuropsychiatric Disorders. Mol. Psychiatry.

[36] Li Y.E., Preissl S., Hou X., Zhang Z., Zhang K., Qiu Y., Poirion O.B., Li B., Chiou J., Liu H. (2021). An Atlas of Gene Regulatory Elements in Adult Mouse Cerebrum. Nature.

[37] Mizrak D., Levitin H.M., Delgado A.C., Crotet V., Yuan J., Chaker Z., Silva-Vargas V., Sims P.A., Doetsch F. (2019). Single-Cell Analysis of Regional Differences in Adult V-SVZ Neural Stem Cell Lineages. Cell Rep..

[38] Baptista P., Andrade J.P. (2018). Adult Hippocampal Neurogenesis: Regulation and Possible Functional and Clinical Correlates. Front. Neuroanat..

[39] Akers K.G., Martinez-canabal A., Restivo L., Yiu A.P., Cristofaro A.D., Hsiang H.L., Wheeler A.L., Guskjolen A., Niibori Y., Shoji H. (2014). Hippocampal Neurogenesis Regulates Forgetting During Adulthood and Infancy. Science.

[40] Eriksson P.S., Perfilieva E., Björk-Eriksson T., Alborn A.M., Nordborg C., Peterson D.A., Gage F.H. (1998). Neurogenesis in the Adult Human Hippocampus. Nat. Med..

[41] Salta E., Lazarov O., Fitzsimons C.P., Tanzi R., Lucassen P.J., Choi S.H. (2023). Adult Hippocampal Neurogenesis in Alzheimer’s Disease: A Roadmap to Clinical Relevance. Cell Stem Cell.

[42] Tosoni G., Ayyildiz D., Bryois J., Macnair W., Fitzsimons C.P., Lucassen P.J., Salta E. (2023). Mapping Human Adult Hippocampal Neurogenesis with Single-Cell Transcriptomics: Reconciling Controversy or Fueling the Debate?. Neuron.

[43] Sell G.L., Barrow S.L., McAllister A.K. (2024). Glutamate Signaling and Neuroligin/Neurexin Adhesion Play Opposing Roles That Are Mediated by Major Histocompatibility Complex I Molecules in Cortical Synapse Formation. J. Neurosci..

[44] Cheung A., Konno K., Imamura Y., Matsui A., Abe M., Sakimura K., Sasaoka T., Uemura T., Watanabe M., Futai K. (2023). Neurexins in Serotonergic Neurons Regulate Neuronal Survival, Serotonin Transmission, and Complex Mouse Behaviors. Elife.

[45] Tromp A., Mowry B., Giacomotto J. (2021). Neurexins in Autism and Schizophrenia-a Review of Patient Mutations, Mouse Models and Potential Future Directions. Mol. Psychiatry.

[46] Choi Y.-B., Li H.-L., Kassabov S.R., Jin I., Puthanveettil S.V., Karl K.A., Lu Y., Kim J.-H., Bailey C.H., Kandel E.R. (2011). Neurexin-Neuroligin Transsynaptic Interaction Mediates Learning-Related Synaptic Remodeling and Long-Term Facilitation in Aplysia. Neuron.

[47] Liakath-Ali K., Polepalli J.S., Lee S.-J., Cloutier J.-F., Südhof T.C. (2022). Transsynaptic Cerebellin 4-Neogenin 1 Signaling Mediates LTP in the Mouse Dentate Gyrus. Proc. Natl. Acad. Sci. USA.

