# Supplementary material for: Immaturity-Dependent Hippocampal Neurogenic Promotion and Fate Shift by Low-Dose Propofol in Neonatal Mice Revealed Through Single-Nuclei RNA-Sequencing
Source: Biomedicines. 2025 Nov 18;13(11):2806. doi: 10.3390/biomedicines13112806 (PMC12650273; doi:10.3390/biomedicines13112806)
Supplement: Supplementary file 1 [file biomedicines-13-02806-s001.zip › biomedicines-3887309-supplementary.pdf]

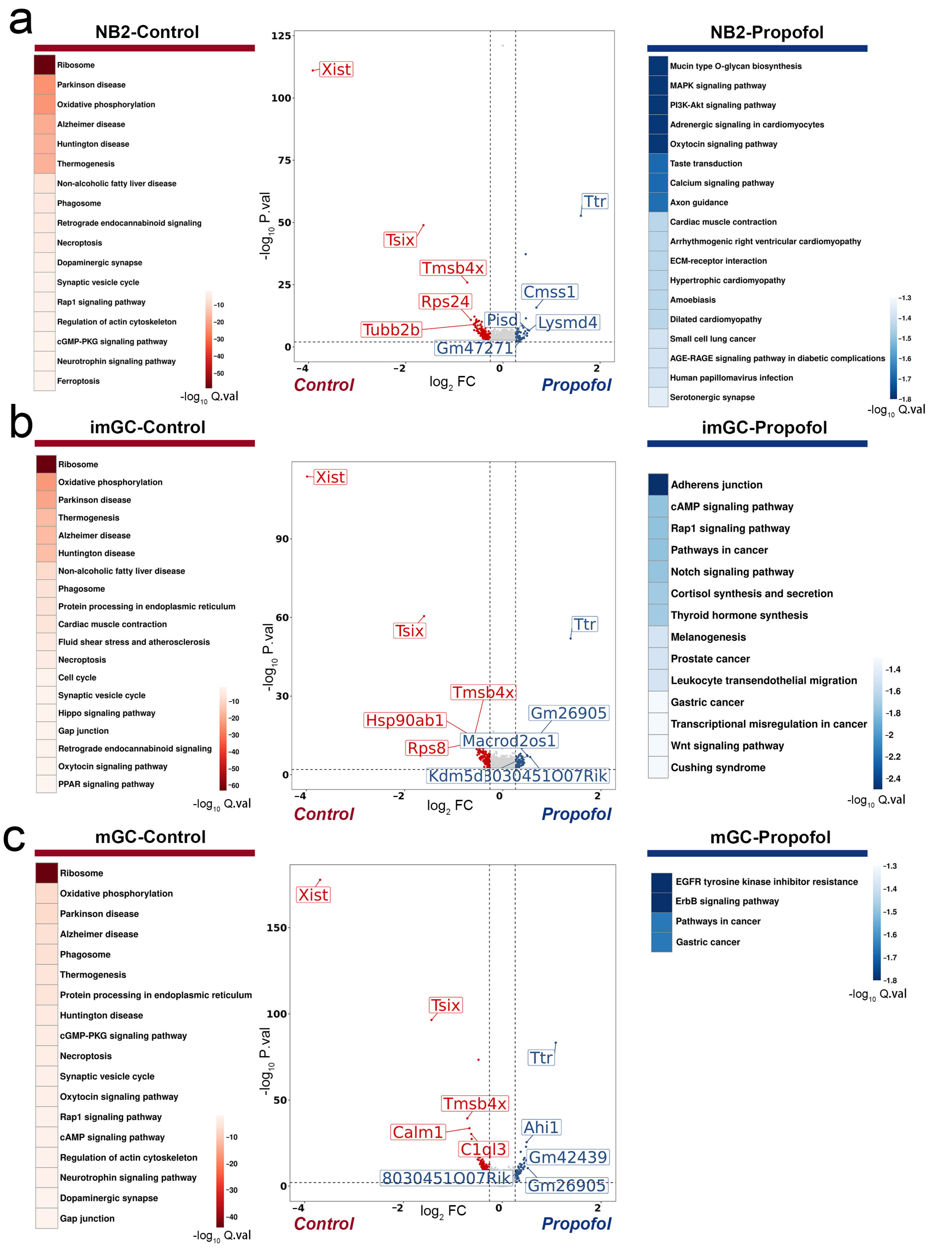

Figure S1. Low-dose propofol regulates synaptic plasticity pathways along the granule maturation lineage: (**a**–**c**) Volcano dot plots and significantly different GO enrichment categories for DEGs enriched in Control (red) and L-Propofol (blue) groups in late granule lineage (NB2 in (**a**), imGC in (**b**), mGC in (**c**)) after L-Propofol treatment. In the volcano plots (**a**–**c**), the vertical dashed lines indicate the $\log_2$ fold change threshold ($|\text{Log}_2\text{FC}| \geq 0.26$), and the horizontal dashed line indicates the adjusted p-value cutoff (Bonferroni-corrected $p < 0.01$).
